# Coordinated variation in root and leaf functional traits of *Hippophae rhamnoides* treated at different stump heights in feldspathic sandstone areas of Inner Mongolia

**DOI:** 10.3389/fpls.2023.1104632

**Published:** 2023-02-14

**Authors:** Lu Liu, Yuefeng Guo, Xiaoyu Liu, Yunfeng Yao, Wei Qi

**Affiliations:** ^1^ College of Desert Control Science and Engineering, Inner Mongolia Agricultural University, Hohhot, China; ^2^ Inner Mongolia Autonomous Region Water Conservancy Development Center, Hohhot, China

**Keywords:** leaves, fine root, plant economics spectrum, stump height, conservation

## Abstract

This study was aimed to clarify the effects of stumping on root and leaf traits as well as the tradeoffs and synergies of decaying *Hippophae rhamnoides* in feldspathic sandstone areas, and to select the optimal stump height that contributed to the recovery and growth of *H. rhamnoides*. variations and coordination between leaf traits and fine root traits of *H. rhamnoides* were studied at different stump heights (0, 10, 15, 20 cm, and no stumping) in feldspathic sandstone areas. All functional traits of the leaves and roots, except the leaf C content (LC) and the fine root C content (FRC), were significantly different among different stump heights. The total variation coefficient was the largest in the specific leaf area (SLA), which is therefore the most sensitive trait. Compared to non-stumping, SLA, leaf N content (LN), specific root length (SRL) and fine root N content (FRN) all improved significantly at stump height of 15 cm, but leaf tissue density (LTD), leaf dry matter content (LDMC), leaf carbon to nitrogen ratio (LC : LN), fine root tissue density (FRTD), fine root dry matter content (FRDMC) and fine root carbon to nitrogen ratio (FRC : FRN) all decreased significantly. The leaf traits of *H. rhamnoides* at different stump heights follow the leaf economic spectrum, and the fine roots show a similar trait syndrome to the leaves. SLA and LN are positively correlated with SRL and FRN and negatively with FRTD and FRC : FRN. LDMC and LC : LN are positively correlated with FRTD and FRC : FRN, and negatively correlated SRL and RN. The stumped *H. rhamnoides* changes to the ‘rapid investment–return type’ resource trade-offs strategy, and the growth rate is maximized at the stump height of 15 cm. Our findings are critical to the prevention and control of vegetation recovery and soil erosion in feldspathic sandstone areas.

## Introduction

The feldspathic sandstone zones of Inner Mongolia are one of the regions with the most severe soil erosion on the Loess Plateau. Feldspathic sandstone is characteristic of a low degree of diagenesis and can be easily invaded to form sand. The construction of artificial vegetation is an important measure of eco-environmental rehabilitation in this region. *Hippophae rhamnoides* is a critical soil and water-conserving plant in arid and semi-arid areas. With well-developed roots, it has strong tillering and germinating abilities and can rapidly spread out to produce large biomass. It has excellent soil water conservation abilities and is extremely dominant in the feldspathic sandstone areas of Inner Mongolia (Liu et al., 2022). However, artificial forests of *H. rhamnoides* in this region that grow to 10 years old will suffer a massive decline in growth and a decrease in productivity (Yang et al., 2014b; Cao et al., 2016), indicating that effective conservation is needed at this age. Since stumping will change functional traits, stumped shrubs can compensationarily recover and grow, and are stimulated to grow more sprouts and branches and thereby improve the net photosynthetic rate and primary productivity, changing the resource intake and use strategies of roots, and preventing the decay of shrub forests (Yang et al., 2020; Liu et al., 2022; Liu et al., 2023). The sprouting effect of stumping is affected by multiple factors, including the controllable stump height (Giambalvo et al., 2011; Langworthy et al., 2019).

Functional traits are used as the response indices of plants to environmental changes and can characterize the survival and resource utilization strategies of plants under unfavourable conditions (Dyer et al., 2001; Reich et al., 2003). The functional traits of leaves play a critical role in the assimilation of carbon, the relationships of moisture, and the energy balance of plants (Ackerly et al., 2002), and the traits of roots decide the absorption of nutrients and moisture that are essential for the survival and growth of plants (McCormack et al., 2015; Sun et al., 2018). However, existing research on plant functional traits is focused on forests and grasslands (He et al., 2008; Hosseini et al., 2019; Cui et al., 2022), and the root and leaf traits of *H. rhamnoides* before and after stumping in feldspathic sandstone areas are still unclear.

The spectrum theory of plant economics (Wright et al., 2004; Freschet et al., 2010) holds that there are associations in functional traits, biomass, construction consumption, and resource absorption between the aboveground and underground parts of plants living under the restrictions of environmental selection and biological–physical factors. This theory also demonstrates the tradeoff between rapid acquisition and resource saving for plants (Reich et al., 1999; Osnas et al., 2013). Apparently, there is a tradeoff and synergy among the functional traits of the leaves. Unlike the leaf trait syndrome, the traits of fine roots belong to be a more complex multidimensional economic space that reflects the diverse evolution pressures and tradeoffs in the underground part (Kong et al., 2014; Xia et al., 2021; Ning et al., 2022). The large specific root length, small diameter and low tissue density of fine roots are related to the high respiration rate and high turnover rate, and such trade-off is similar to the economic spectrum of leaves (Caplan et al., 2019; Laughlin et al., 2021). Other research shows that the traits of fine roots are mutually independent to a large extent. Furthermore, it is unclear whether the trait syndromes of the leaves and roots in *H. rhamnoides* exist before and after stumping.

To achieve the economic spectrum, the traits of different organs (e.g., leaves, roots) have to coordinate in accordance with the evolution and biophysical restraints (Reich, 2014; Weemstra et al., 2016). As reported, similar leaf and root traits are interrelated among species in the grasslands of the Inner-Mongolia Plateau or the Qinghai-Tibetan Plateau (Geng et al., 2014). Compared to forest species, species in arid areas tend to acquire underground resources rather than ground resources (Liu et al., 2010; Bardgett et al., 2014). However, it is unknown whether the existing theory applies to H. *rhamnoides* grown in feldspathic sandstone areas. In addition, there is little research on coordination in chemical tissue traits and morphological traits between the leaves and fine roots of *H. rhamnoides* in feldspathic sandstone areas.

For these reasons, in this study targeted at *H. rhamnoides* grown in feldspathic sandstone areas of Inner Mongolia, we analyzed the variation traits of roots and leaves at different stump heights as well as the tradeoffs and synergies between them. Specifically, we tested three hypotheses: (1) the leaf-trait syndrome in *H. rhamnoides* at different stump heights is in line with the global prediction of LES and is in parallel with the root-trait syndrome; (2) functional traits related to nutrient content and resource absorption are closely coordinated between leaves and fine roots; (3) stumped *H. rhamnoides* change to the ‘rapid investment–return type’ resource trade-off strategy.

## Materials and methods

### Experimental sites

**Figure 1:**
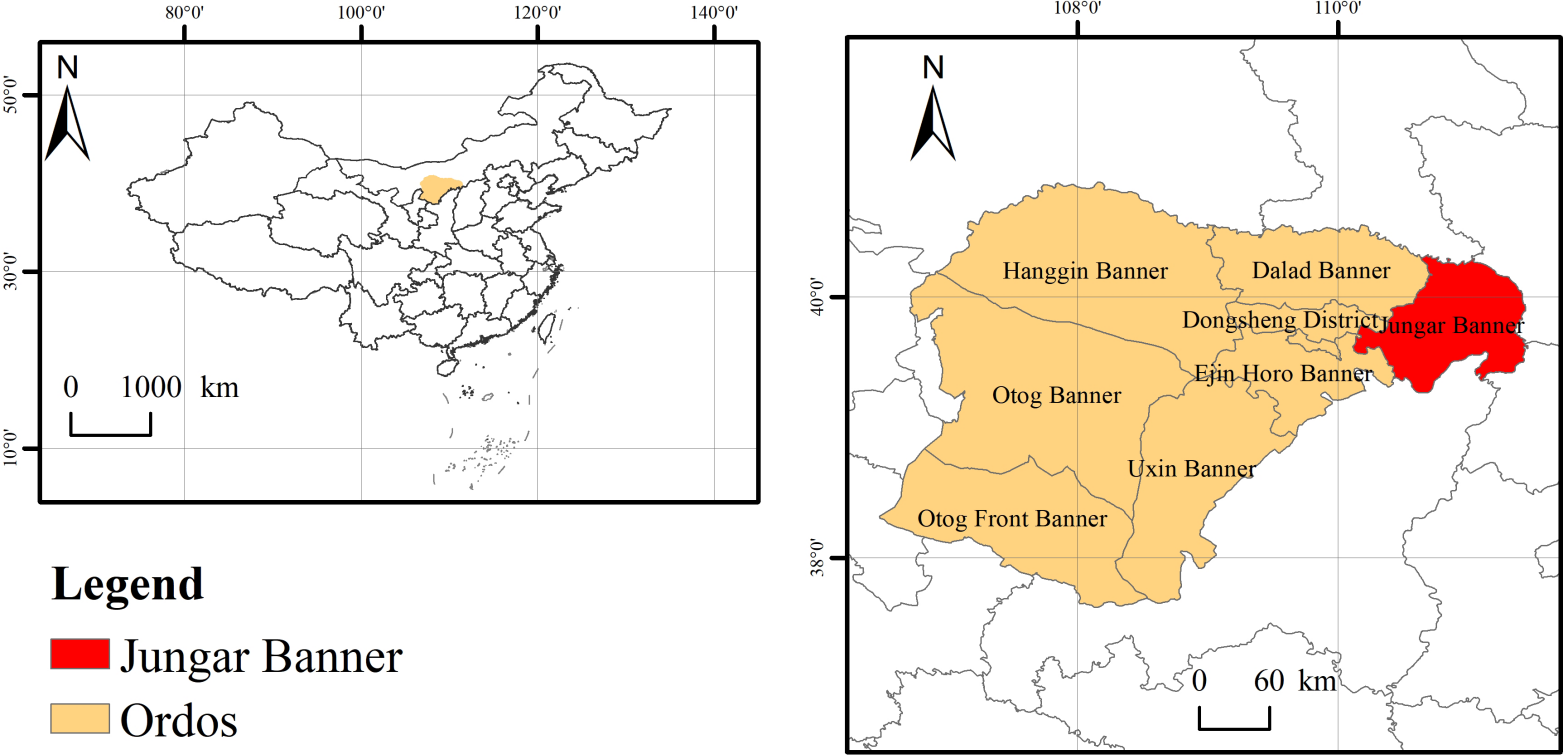
Geographical positions of the study area.

The study area is located in the soil-water conservation science and technology demonstration zone in the feldspathic sandstone zone in Nuanshui Village Jungar Banner, Ordos, Inner Mongolia (**Figure 1**). This region (39°42’N -39°50’ N, 110°25’E -110°48’ E, 96 km^2^) has complex terrains with extensive gullies and fluctuating girders, and suffers soil erosion and soil-water loss. It has an average altitude of 800 - 1590 m, a precipitation of 400 mm (concentrated in July and August), a duration of sunlight duration above 3000 d, an evaporation of 2093 mm, and a temperature of 6.2-8.7°C on the annual average. The vegetation of this region is dominated by artificial vegetation, including *H. rhamnoides*, *Pinus tableulaeformis*, *Caragana korshinskii*, *Medicago sativa* and *Prunus sibirica*.

### Experimental design

**Figure 2:**
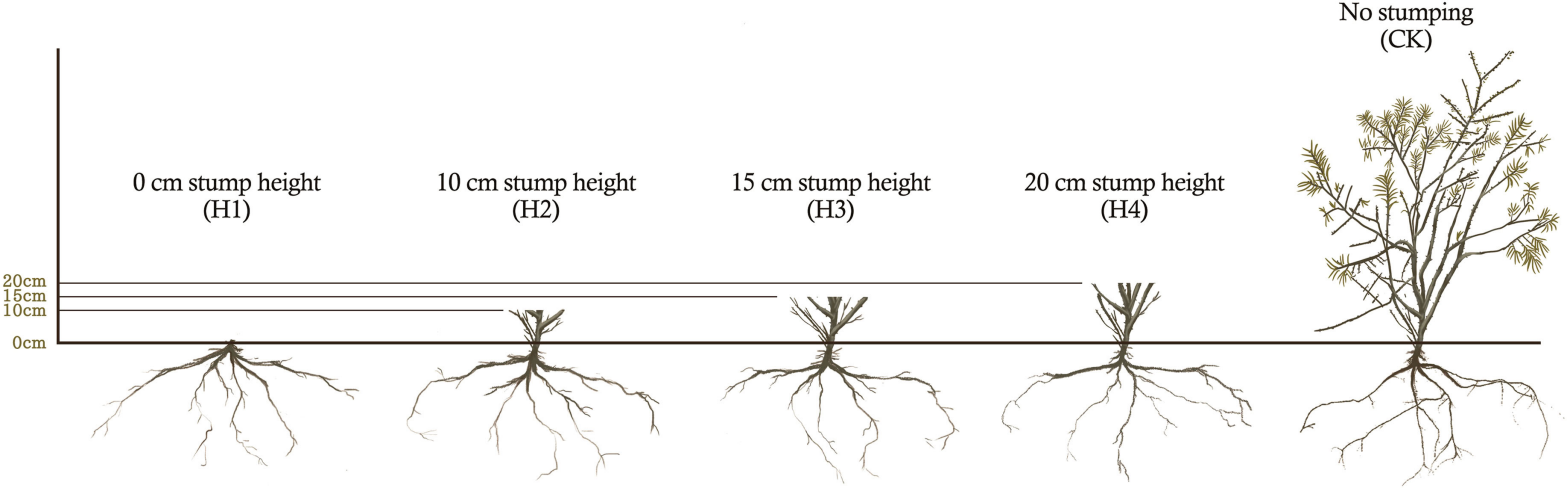
Schematic diagram of different stump heights treatment of *H. rhamnoides*.

In the demonstration zone, we chose *H. rhamnoides* plantation land that had basically consistent site conditions and forest compositions and were in the decaying stage as the experimental site, which were under the northwest slope face at slope of 4°. On the same slope face, *H. rhamnoides* were planted at the row space 0f 2m × 4m, and its tree age was 10 years. *H. rhamnoides* were stumped at early March 2020 in the experimental site. The stump heights (the stumping distance from the ground) were 0, 10, 15 and 20 cm, which corresponded to the treatments H1, H2, H3 and H4, respectively (**Figure 2**). A stump-free plantation site was established as a control (CK). All treatment sample plots were 50m×50m in area. Each treatment was carried out in triplicate. Stumping was performed using electric saws and pruning shears, which ensured that the incisions were flat and smooth without burrs. The overall stumping mode was adopted. To decrease plant moisture dissipation, we painted stumped sites after stumping.

### Sample collection and processing

The leaf and fine root traits in five samples from the plots treated at different stump heights were measured in the middle growing season (middle June to lower August) of 2022 (The growth of *H*. *rhamnoides* at different stump heights was listed in **Table 1**). A sample involved at least five representative and healthy plants under basically consistent growth status. From each plant, 10 healthy leaves in medium size were collected and used to measure morphology, C and N concentrations. Fine roots were collected by the digging method. The weeds and litter around the basal of each chosen sample were cleaned away. After the surface soils were mostly cleaned, the peri-root soils were carefully cleared away to find the main roots. When root branches were encountered in the growing direction of the main roots, we further dug along the branch roots until we reached the end of the root system. During sampling, the loss of terminal low-level roots was avoided to ensure root completeness. Then fine roots in diameter smaller than 2 mm were chosen (Cornelissen et al., 2003; Mitchell et al., 2017).

**Table 1:** Growth of *H. rhamnoides* at different stump heights.

Stump height	Average tree height (cm)	East–west canopy size (cm)	South–north canopy size (cm)
H1	82.83 ± 1.88d	61.32 ± 2.03d	55.65 ± 1.93d
H2	96.54 ± 1.79c	71.99 ± 2.41bc	67.54 ± 1.84c
H3	102.01 ± 2.21b	77.41 ± 2.06b	73.25 ± 2.12b
H4	86.58 ± 2.10d	66.73 ± .2.29cd	63.78 ± 2.58c
CK	110.45 ± 1.73a	93.28 ± 2.09a	87.76 ± 2.07a

Values are means ± SD. Significant differences are indicated by different lowercase letters at *p* < 0.05.

The roots collected as they were put in labeled preservation bags, which were then placed in a refrigerator at 2-3° C on the same day and taken back to our laboratory. The leaves and fine roots were washed with deionized water and scanned using an 11000XL scanner (Epson, Tokyo, Japan). Together with WinRHIZO Pro 2012b (Regent Instruments Inc., Quebec City, Canada), leaf areas, leaf volumes, total fine root length, and fine root volumes were measured. Subsequently, the leaves and roots in each sample were placed in water and stored away from light at 4°C for 24 h (Ning et al., 2022). After the water was saturated, the surface water of the leaves or fine roots was sucked using absorbent paper, and then the saturated fresh weight of the leaves or fine roots was measured. Then the leaves and fine roots were dried at 60°C for 48 h, and the dry weight of the leaves and the dry weight of the fine roots were monitored at constant weight (Cui et al., 2019).

Then the specific leaf area (SLA), leaf dry matter content (LDMC), leaf tissue density (LTD), specific root length of fine roots (SRL), fine root dry matter content (FRDMC), and fine root tissue density (FRLTD) were computed. SLA and SRL are the ratio of area to dry weight of leaves and the ratio of area to dry weight of fine roots respectively. LDMC and FRDMC are the ratio of dry weight to saturated water weight of leaves and the ratio of dry weight to saturated water weight of fine roots, respectively. LTD and FRTD stand for the ratio of dry weight to volume of leaves, and the ratio of dry weight to volume of fine roots respectively (Jiang et al., 2021). The dried leaves and fine roots were ground into powder using a PULVERISETTE 5 high flow ball milling system (Fritsch, Munich, Germany), which was passed through a 0.149 mm sieve for chemical analysis. Carbon and nitrogen content in fine roots and leaves were measured using a Vario MACRO cube elemental analyzer (Elementar, Hanau, Germany), and the carbon and nitrogen ratios in roots and leaves were determined.

### Data processing

Data were analyzed in SPSS 26.0. The data of leaves and fine roots were sent to descriptive statistics and analysis of the variation coefficient (variation coefficient =standard deviation/mean value ×100%). Differences in the traits of leaves and fine roots of *H. rhamnoides* among different stump heights were statistically analyzed by one-way analysis of variance (ANOVA). Significance was tested using Fisher’s least significant difference method at the level *p* < 0.05. The leaf and fine root variation characteristics at different stump heights, the Pearson heatmaps of fine roots and leaves, and the PCA of root and leaf coordination were plotted in Origin 2021.

## Results

### Variation of leaf and fine root traits

**Figure 3:**
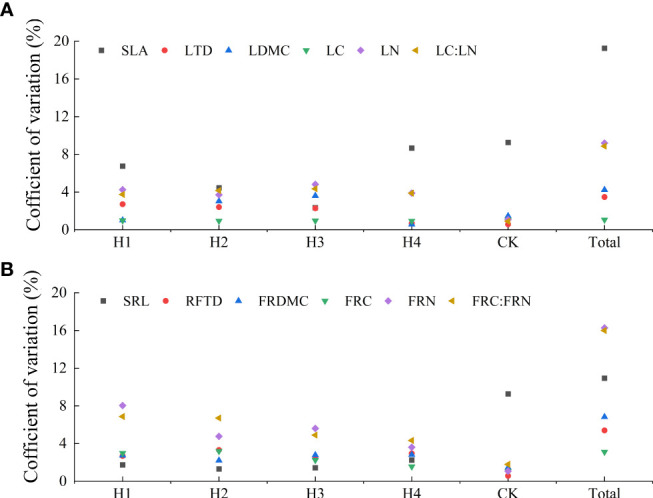
Variation traits of leaves **(A)** and fine roots **(B)** at different stump heights. Specific leaf area (SLA), Leaf tissue density (LTD), Leaf dry matter content (LDMC), Leaf C content (LC), Leaf N content (LN), Leaf C:N ratio (LC : LN), Specific fine root length (SRL), Fine root tissue density (FRTD), Fine root fry matter content (FRDMC), Fine root C content (FRC), Fine root N content, Fine root C:N ratio (FRC : FRN).

Neither leaf C content (LC) nor fine root carbon content (FRC) was significantly different among different stump height treatments (*p* > 0.05) (**Table 2**; **Figure 3**). SLA, LTD, LDMC, LC, leaf nitrogen content (LN), leaf carbon to nitrogen ratio (LC : LN), SRL, FRID, FRMDC, FRC, fine root nitrogen content (FRN) and fine root carbon to nitrogen ratio (FRC : FRN) were very significantly different between treatments (*p* < 0.001).

**Table 2:** Analysis of variance of leaf and fine root functional traits.

traits	traits	df	SS	MS	F	P
Specific leaf area (cm^2^)	SLA	4	35618.7464	8904.6866	54.576	0.0001
Leaf tissue density (g cm^3^)	LTD	4	0.0003	0.0001	14.395	0.0001
Leaf dry matter content (g g^-1^)	LDMC	4	0.0028	0.0007	17.021	0.0001
Leaf C content (g kg^-1^)	LC	4	115.5557	28.8889	1.227	0.3311
Leaf N content (g kg^-1^)	LN	4	61.2188	15.3047	28.132	0.0001
Leaf C:N ratio (g kg^-1^)	LC : LN	4	69.4748	17.3687	10.489	0.0001
Specific fine root length (cm g^-1^)	SRL	4	2100.1523	525.0381	229.688	0.0001
Fine root tissue density (g cm^-3^)	FRTD	4	1.5629	0.3907	17.022	0.0001
Fine root fry matter content (g g^-1^)	FRDMC	4	0.0210	0.0052	30.336	0.0001
Fine root C content (g kg^-1^)	FRC	4	1526.1329	381.5332	2.503	0.0749
Fine root N content (g kg^-1^)	FRN	4	26.7328	6.6832	40.813	0.0001
Fine root C:N ratio (g kg^-1^)	FRC : FRN	4	2303.5383	575.8846	30.928	0.0001

degree of freedom (df), sum of squares (SS), mean square (MS), statistic (F), significant (p).

The total coefficient of variation among different stump heights was 1.05%–19.26%. Total coefficients of variation in SLA, FRN, FRC : FRN, and SRL were greater than 10%, with a maximum in SLA (19.26%). The total coefficients of variation in LN, LC : LN, RDMC, RTD, LDMC, LTD, FRC, and LC were all larger than 10%, with the minimum values of 1.05% in LC and 3.22% in FRC. The total coefficient of variation among different treatments was classified as SLA > FRN > FRNC : FRN > SRL > LN > LC : LN > FRDMC > FRTD > LMDC > LTD > FRC > LC. The coefficients of variation in all indices of H1, H2, H3, H4 and CK were all lower than 10% (**Figure 3**).

**Figure 4:**
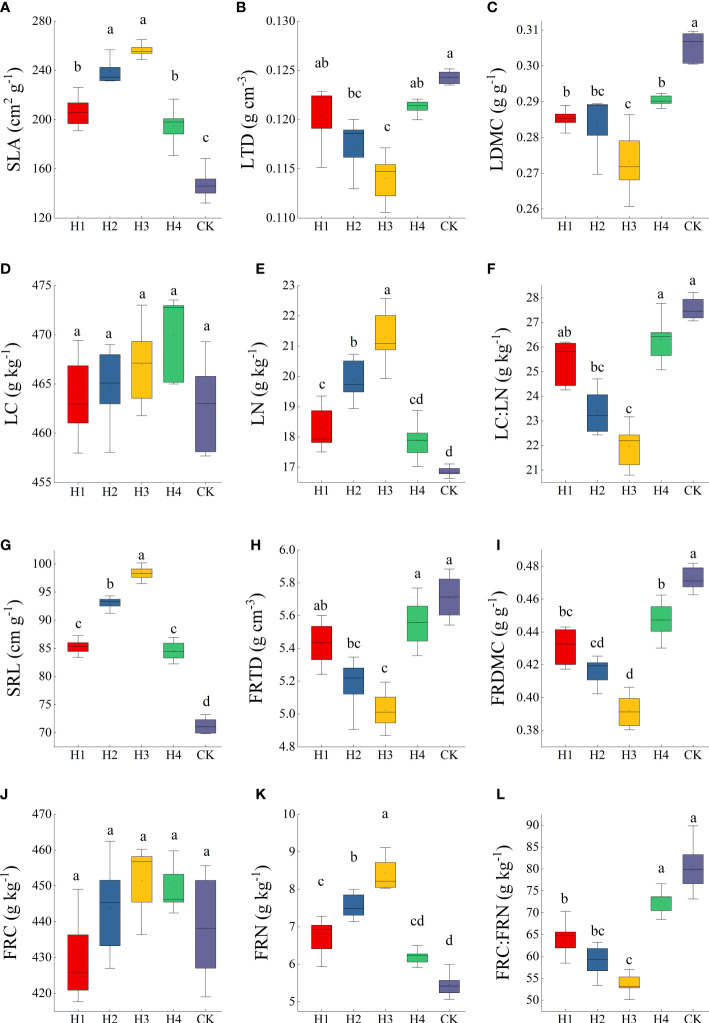
The traits of leaves **(A–F)** and fine roots **(G–L)** at different stump heights. Error bars represent ± SE of the mean. Significant differences are indicated by different lowercase letters at *p* < 0.05. (All abbreviations are shown in (**Figure 3**).

The SLA, LTD, LDMC, LC, LN, LC : LN, SRL, FRID, FRMDC, FRC, FRN, and FRC : FRN were all significantly different between H2 and H3 (*p* < 0.05). Compared to CK, SLA, LN, SRL and FRN improved significantly after stumping, but LTD, LDMC, LC : LN, FRTD, FRDMC and FRC : FRN significantly decreased. SLA, LN, SRL and LN ranked as H3 > H2 > H1 > H4 > CK. The changing rules of LTD, LDMC, LC : LN, FRTD, and FRDMC were the opposite and ranked as H3 < H2 < H1 < H4 < CK (**Figure 4**).

### Trait correlations in leaves and fine roots

**Figure 5:**
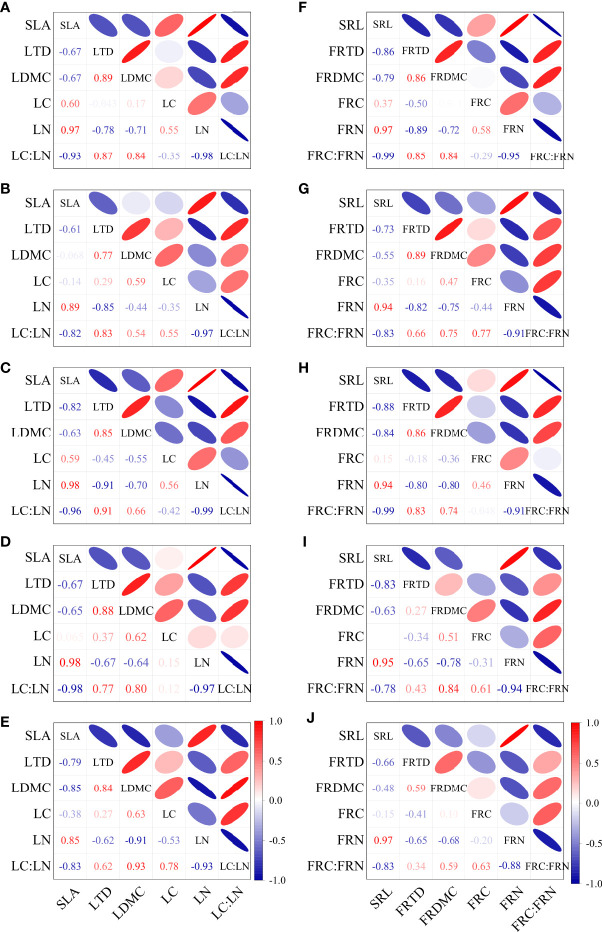
Correlations of leaf traits and of fine root traits at different stump heights **(A–E)**. Correlations of leaf properties at 0, 10, 15, 20 cm and no-stumping **(F–J)**. Correlations of fine root properties at 0, 10, 15, 20 cm and without stumping. (All abbreviations are shown in **Figure 3**).

The leaf traits and the fine root traits were both significantly correlated among different stump heights (**Figure 5**). SLA in CK was negatively correlated with LTD, LDMC, and LC : LN, and positively correlated with LN. LDMC in CK was positively correlated with LTD and LC : LN (*p* < 0.05), and very significantly negatively correlated with LN (*p* < 0.01).The LN and LC : LN in CK were very significantly negatively correlated (*p* < 0.01, **Figure 5E**). The correlations of SLA with LTD and LDMC were weakened after stumping, but the correlation between SLA and LN or LC : LN was enhanced. In particular, the SLA in H1, H3 or H4 was highly significantly correlated positively with LN (*p* < 0.01) and negatively with LC : LN (both *p* < 0.01). After stumping, LTD was positively correlated with LDMC and negatively with LN (*p* < 0.05), and LN was very significantly positively correlated with LC : LN (*p* < 0.01, **Figures 5A–D**).

SRL in CK was negatively correlated with RTD and FRC : FRN (*p* < 0.05), and was very significantly positively correlated with FRN (*p* < 0.01). FRN and FRTD in CK were positively correlated with FRDMC (*p* < 0.05), and were very significantly negatively correlated with FRC : FRN (*p* < 0.01, **Figure 5J**). SRL after stumping was negatively correlated with RTD and FRC : FRN (*p* < 0.05), but SRL in H1 or H3 was very significantly negatively correlated with FRC : FRN (*p* < 0.01). The FRN and FRTD after stumping were both positively correlated with FRDMC (*p* < 0.05), and the FRTD and FRDMC of H1, H2, H3 were positively correlated with FRC : FRN (*p* < 0.05).The FRN was still very significantly negatively correlated with FRC : FRN after stumping (*p* < 0.01, **Figures 5F–I**), and the correlation was unchanged. Clearly, the correlations between leaf traits and between fine root traits differ at different stump heights.

### Coordination between leaf and fine root traits

**Figure 6:**
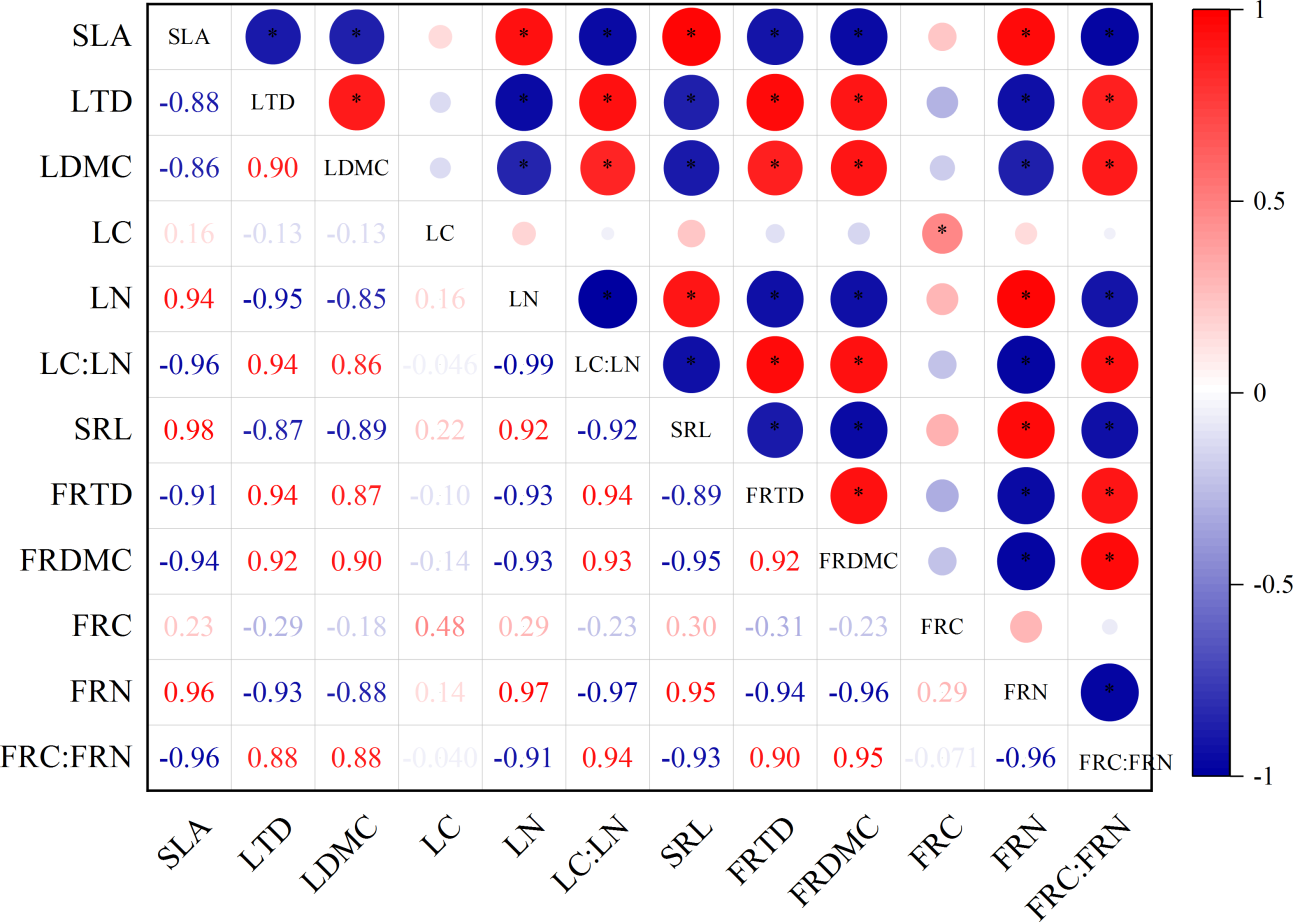
Correlations between leaves and fine roots. (All abbreviations are shown in (**Figure 1**).

There was a significant correlation between the leaf and fine root traits of *H*. *rhamnoide* (**Figure 6**, *p* < 0.05).SLA is negatively correlated with LTD, LDMC and LC : LN, and positively with LN. LTD is positively correlated with LDMC and LC : LN, and negatively correlated with LN. LN and LC : LN are very significantly positively correlated (*p* < 0.01). SLA and LN are both positively correlated with SRL and FRN and negatively correlated with FRTD and FRC : FRN. LDMC and LC : LN are positively correlated with FRTD and FRC : FRN respectively, and are both negatively correlated with SRL and RN. The correlations of the fine root traits are similar to those of the leaves. SRL is negatively correlated with FRTD and FRC : FRN, and very significantly positively with FRN (*P* < 0.01). Both FRTD and FRDMC are negatively correlated with FRN and positively with FRC : FRN. FRN and FRC : FRN are negatively correlated.

**Figure 7:**
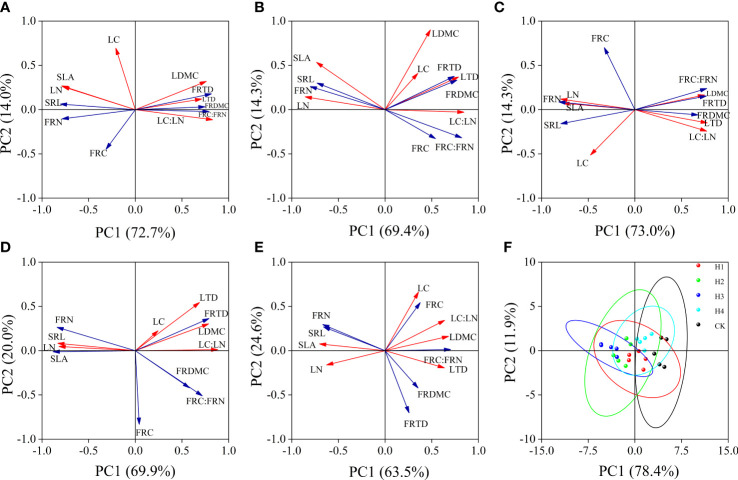
Coordination of root and leaf at different stump heights **(A–E)**. Principal component analysis of leaves and fine roots at 0, 10, 15, 20 cm and without stumps respectively. **(F)** is the analysis of the principal component of the leaves and fine roots at different stump heights. (All abbreviations are shown in **Figure 3**).

The accumulative contribution rates of variance on axis 1 and axis 2 in the PCA at different stump heights were both above 80%, which can well reflect the relationship between the functional traits of leaves and fine roots. CK PC1 is a structural axis decided mainly by SLA, LN, LDMC, FRC : FRN, and LTD, and its PC2 is defined by LC, FRC, and FRTD. The closest correlations in CK were found between LC : LN and LN, between FRN and SRL, and between FRC : FRN and SLA (**Figure 7E**). The indices in H1, H2, H3 and H4 after stumping did not change much on the PC1 axis, but LC and FRC axis 2 in all indices and the correlations between root and leaf traits were enhanced. In particular, the associations of SLA or SRL with LN, LC : LN, FRN, and FRC : FRN were closer (**Figures 7A–D**). Although the confidence groups overlapped slightly at different stump heights, the root and leaf traits after different treatments aggregated and were mutually separated. The CK root and leaf traits were mainly distributed in the right half of axis 2, and the root and leaf traits of H1, H2, H3 and H4 shifted along axis 2 from right to left, and H3 was mainly distributed in the right half of PC2 (**Figure 7F**).

## Discussion

### Variation in root and leaf morphological traits

The results showed that stumping significantly affected the morphological traits of both roots and leaves (**Table 2**). The coefficients of variation in the functional morphological traits of the roots and leaves at different stump heights fell within 3.47%–19.25%, which were all smaller than 20%. The coefficients of variation in SLA and SRL were large and very significantly different among different stump heights. These results indicate that these functional traits are very sensitive to stumping. The tissue densities and dry matter content of both roots and leaves are relatively stable variables on the resource acquisition axis, and thus the coefficients of variation are small (**Figure 3**).

According to leaf and root economics spectra (LES and RES respectively), SLA and SRL are two key traits reflecting the resource strategies of plants (Wright et al., 2004; Cheng et al., 2016). Specifically, the species with larger SLA had higher LN, leaf P content, faster photosynthetic rate, shorter leaf longevity, and lower LTD, which indicates the resource-acquisitive strategy. On the contrary, the species with lower SLA were conservative. The fine roots with large SRL, small diameter, low RTD and high N content are related to the low construction cost, fast respiration rate and high turnover rate. This pattern is similar to that of leaf trait correlations (Caplan et al., 2019; Reich, 2014).

The specific leaf area reflects the light receiving and capturing area per unit of leaf dry weight and indicates the abilities of plants to use environmental resources and to store the acquired resources. It is closely related to the rate of assimilation rate and the survival countermeasures of plants (Fajardo and Siefert, 2016). The specific fine root length is an important morphological structure that decides the water and nutrient absorption capacity of roots (Wang et al., 2019; Wang et al., 2020). Generally, the specific leaf area is larger in a resource-rich environment. Plants subjected to nutrient restriction or interspecific competition will increase the specific root length and root specific surface area to improve the acquisition capacity or competitiveness of nutrients (Kraft and Ackerly, 2010; Yang et al., 2014a). A decrease in tissue density or dry matter content in roots or leaves can rapidly accelerate plant growth turnover, so the loss of moisture and nutrients will decreased, thus improving the efficiency of use of moisture and nutrient and improving the defense force (Laughlin et al., 2021; Ning et al., 2022).

Our results showed that compared to CK, the SLA and SRL of *H. rhamnoides* were increased significantly after stumping, and LTD, LDMC, RTD, and RDMC were reduced (**Figure 4**), which are basically consistent with previous research. The reasons are that during compensationary recovery and growth after stumping, the resource acquisition conditions of *H. rhamnoides* are altered, as the cost of leaf construction is lowered. Consequently, the evaporation of vegetation decreases and the root branching ability and the soil nutrient absorption ability are enhanced, which promotes *H. rhamnoides* to adjust the adapting strategy to enhance its viability and to provide moisture and nutrients for the growth and metabolism of plants (Yuan et al., 2020; Liu et al., 2022), forming a growing strategy of high SLA and SRL and low LTD, LDMC, RTD and RDMC. This validates our first hypothesis. SLA, LN, SRL and LN all rank as H3 > H2 > H1 > H4 > CK. The changing rules of LTD, LDMC, LC : LN, FRTD, and FRDMC are the opposite and rank as H3 < H2 < H1 < H4 < CK (**Figure 4**).This compensation recovery and growth strategy was optimized at the stump height of 15 cm, above which the promoting effect was lowered.

### Variation in chemical tissue traits in roots and leaves

The C element is the substrate and energy source of various physiological-biochemical processes of plants, and is the most important step to connect the external inorganic environment and organisms. C is also the basic framework of all organisms and is closely related to the photosynthesis and respiration of plants (Xu et al., 2018). N, a basic nutrient element of plants, and is an important compositional element and adjustment substance of diverse proteins and genetic materials (Cui et al., 2022). The C:N reflects the carbon assimilation ability of plants during the absorption of nutrient elements and indicates the use efficiency of nutrient elements. Generally, low C:N suggests that a plant grows fast (Hu et al., 2022).

We found that stumping significantly affected both N and C:N of leaves and roots, but leaf C contents were not significantly different among different stump heights (**Table 2**), and the root and leaf C contents were highly stable (**Figure 3**). This was because the organic carbon in the organs of plants is usually not directly involved in production activities, but acts as skeleton to provide plant activities with energy, and thus the organic carbon content *in vivo* is large and stable with low variation (Wang et al., 2018). Our results showed that the C : N was always larger in roots than in leaves at all stump heights, and the C : N in both leaves and roots was not significantly different among different stump heights (**Figure 4**), indicating the rate of N utilization is larger in roots than in leaves. The C and N contents in leaves are always larger than in roots regardless of the stump height, which is because the efficiency of C and N use of leaves is lower than that of roots. Hence, when cell division occurs rapidly due to the rapid growth of the leaves, the leaves demand largely for nutrient. Consequently, the C produced from photosynthesis gradually accumulates, and the roots transport more N to leaves, which is used in the synthesis of proteins and nucleic acids (Craine, 2006; Morales et al., 2015), so the C and N contents in the leaves of *H. rhamnoides* are higher.

Leaf nitrogen is a key factor for photosynthetic material metabolism and plant growth, and is an important component for the synthesis of chlorophylls and relevant photosynthetic proteins. Leaves with high N contents usually have fast photosynthetic rate (Jaikumar et al., 2013). Our results showed the leaf and root N contents after treatments H1, H2, H3, and H4 were all significantly larger compared to the CK (**Figure 4**). This was because the metabolism of roots was enhanced after the stumping, which promoted the nitrogen fixation ability of root nodules and improved the absorption and transportation of nitrogen nutrient elements by fine roots from the soils. As a result, the nitrogen contents in roots and leaves were significantly improved to maintain the rapid recovery and growth of plants (Yang et al., 2020; Liu et al., 2022; Liu et al., 2023). In addition, leaf area is an indicator of the photosynthesis ability of leaves. A larger leaf area is favorable for the interception of more solar light to produce organic matter (Osnas et al., 2013). Our calculations showed the leaf areas after stumping were significantly larger compared to CK. The increased leaf area and the increase nitrogen transport to leaves through roots after the stumping jointly promoted the efficiency of plant photosynthesis and accelerated the synthesis of abundant chlorophyll and photosynthetic proteins in leaves, which once again increased the leaf N content (Craine, 2006; Morales et al., 2015). This again validates our first hypothesis.

The C: N ratios in the roots or leaves after treatments H1, H2, H3, and H4 were all significantly lower than those without stumping (**Figure 4**), suggesting *H. rhamnoides* at the stump height of 15 cm can grow faster. In all, stumping can not only control the external growing morphology of plants to some extent, but can also indirectly change the internal physiological processes by adjusting the needed resources and environment, which will considerably affect plant growth.

### Root and leaf trait coordination

The functional traits of plants are not mutually independent, but are coordinated or traded off to promote plant growth. Then the correlations among the leaf functional traits were compared between CK and H1, H2, H3 or H4. Commonness was found, as SLA and LN were positively correlated, and LC : LN was negatively correlated with both SLA and LN. It is speculated the coupling between the 2 above traits is the most stable among the leaf functional traits of *H. rhamnoides*.

The correlations of SLA with LTD and LDMC were weaker after the stumping, and the correlations between SLA and LN, and between LC : LN and LN were strengthened (**Figures 5A–E**). These correlations between traits are similar to the findings on leaf functional traits in accordance with the leaf economics spectrum (Wright et al., 2004; Marechaux et al., 2020), which reflects that the associations between the leaf functional traits are universal. However, we found the correlations among leaf morphological traits were weakened after the stumping, and the correlations with chemical characteristics of the tissue were enhanced. Adjusting leaf morphological structure, growing pattern and nutrient element allocation strategy after stumping will alter the correlations between traits, so the stumped plants can rapidly recover the lost aground branches, which will improve the nutrient absorbing capacity of roots and the nutrient transport ability to leaves (Yang et al., 2020; Liu et al., 2022). The morphological traits of leaves and the absorption of nutrient resource by leaves are directly and closely associated (Ackerly et al., 2002; Wright et al., 2004). This is also the reason for the increased. correlations between the leaf morphological traits and chemical tissue traits after the stumping. The change between morphological traits may be indirect after the chemical tissue traits of leaves affect a certain morphological trait. Therefore, during the compensationary recovery and growth of leaves, the correlations between the morphological traits of the leaves are weakened.

We found the correlations between fine root traits of *H. rhamnoides* were similar to the correlations between leaf traits, especially SRL and SLA that reflect the resource acquisition abilities of fine root and leaves respectively (Reich, 2014; Laughlin et al., 2021). The results showed that SRL was negatively correlated with RTD, and FRC : FRN, and very significantly positively correlated with FRN. FRTD and FRDMC were both positively correlated with FRN. FRN and FRC : FRN were very significantly negatively correlated (**Figures 5F–J**). All of these results basically consistent with previous studies. The correlations of root morphological traits after the stumping with FRN, and FRC : FRN were enhanced, which is consistent with the correlations between leaf traits after the stumping.

In a dry environment, the functional traits related to nutrition contents and resource absorption are closely coordinated between leaves and fine roots. This is because the moisture and nutrient restrictions for plant growth require that the functions of fine roots (namely, moisture and nutrient absorption) must match the functions of leaves (namely photosynthesis and transpiration) (Carvajal et al., 2019). Our root and leaf correlation analyzes prove this view: SLA and LN are both positively correlated with SRL and FRN, and negatively correlated with FRTD and FRC : FRN. LDMC and LC : LN are positively correlated with FRTD and FRC : FRN respectively, and negatively correlated with SRL and RN (**Figure 6**). Namely, nutrient absorption by the ground and underground organs is strongly associated. These results further prove that the aground traits and underground traits of *H. rhamnoides* are coordinated to some extent. Our results are consistent with previous studies on temperate grasslands (Craine et al., 2005), forests (Holdaway et al., 2011) and lawn (Liu et al., 2010; Zhao et al., 2016). This validates our second hypothesis.

According to the plant economics spectrum theory, functional traits of plants are important indices for measuring environmental resource trade-off strategies of plants (Reich et al., 2003; Freschet et al., 2010). When LTD, LDMC, RTD, RDMC, and C: N are low, SLA and SRL are large, and when the N contents in roots and leaves are large, the plants usually have a rapid photosynthetic rate and growth rate and become the ‘rapid investment–return’ type; otherwise, they approach the ‘slow investment –return’ type (Jiang et al., 2021; Pérez-Ramos et al., 2012). Compared with CK (**Figure 7E**), the LTD, LDMC, FRTD, FRDMC, LC: LN, and FRC : FRN significantly decreased, SLA, and SRL significantly rose, and LN and FRN were large after the stumping (**Figures 4**, **7A–D**). It is indicated *H. rhamnoides* in an environment with limited resources is indicated to optimally allocate resources between the functional traits of leaves and roots by using a trade-off strategy. Compared with CK, the root and leaf economics spectra of stumped *H. rhamnoides* are closer to one end of ‘rapid investment–return type’ species (**Figure 7F**), which indicates the shift to the resource trade-off strategy of ‘rapid investment–return type’. This validates our third hypothesis. Our findings are significant for the prevention and control of revegetation and soil erosion in feldspathic sandstone areas.

## Conclusions

The variation and coordination between the traits of the roots and leaves were analyzed at different stump heights. The results show that the leaf traits of *H. rhamnoides* at different stump heights agree with the global prediction of LES, and are in parallel with the root-trait syndrome. The *H. rhamnoides* after stumping has a high SLA, SRL, LN, and FRN, and low LTD, LDMC, LC : LN, FRTD, FRDMC, and FRC : FRN. It is indicated that *H. rhamnoides* in an environment with limited resources can optimally allocate resources between functional traits of leaves and roots using a trade-off strategy, and thus *H. rhamnoides* shifts to the resource trade-off strategy of ‘rapid investment-return type’. The coordination between the leaves and the fine roots is stronger in terms of the chemical tissue traits compared to the morphological traits. Generally, stumped *H. rhamnoides* can grow faster compared to unstumped shrubs, and the optimal stump height is 15 cm. Thus, to improve the decaying of *H. rhamnoides* forests in feldspathic sandstone areas, shrubs can be stumped at a height of 15 cm.

## Data availability statement

The original contributions presented in the study are included in the article/supplementary material. Further inquiries can be directed to the corresponding author.

## Author contributions

LL: Conceptualization, Methodology, Writing–original Draft, Visualization, Data curation, Software, Investigation, Formal analysis. YG: Conceptualization, Methodology, Writing –original Draft, Writing–review and editing, Supervision, Project administration. XL: Conceptualization, Methodology, Writing–original Draft, Data curation, Software, Investigation, Formal analysis. YY: Conceptualization, Writing–original Draft, Writing–review and editing, Supervision, Project administration. WQ: Conceptualization, Writing–original Draft, Visualization, Investigation, Formal analysis. All authors contributed to the article and approved the submitted version.
